# Glucose-6-phosphate dehydrogenase deficiency and neonatal indirect hyperbilirubinemia: a retrospective cohort study among 40,305 consecutively born babies

**DOI:** 10.1038/s41372-024-01927-1

**Published:** 2024-03-13

**Authors:** Rajai Rofail Raja Al-Bedaywi, Khalil Mohd Khalil Salameh, Sarfrazul Abedin, Brijroy Viswanathan, Abedalkhalik Ahmad Khedr, Lina Hussain M. Habboub

**Affiliations:** Department of Neonatology, AlWakra hospital, Hamad Medical Corporation, Doha, Qatar

**Keywords:** Paediatrics, Diagnosis

## Abstract

**Background and objective:**

Glucose-6-phosphate dehydrogenase deficiency (G6PDD) being highly prevalent in the Middle East, the primary objective was to estimate the incidence of neonatal jaundice among G6PD-deficient neonates and to explore its association with various risk factors.

**Methods:**

This retrospective cohort study includes 7 years data of neonates diagnosed with G6PDD between 1st January 2015, and 30 September 2022, from Al Wakra Hospital, HMC Qatar.

**Results:**

Among the 40,305 total births, 1013 had G6PDD with an incidence of 2.51%. Of all the G6PDD babies, 24.6% (249/1013) received phototherapy and three babies required exchange transfusion. Statistically significant associations were noted between the need for phototherapy and gestational age, gestational age groups, birth weight, and birth weight groups, but logistic regression analysis showed significant association for phototherapy only with the gestational age group.

**Conclusion:**

Universal screening and proper follow-up is essential for G6PDD as it plays crucial role in neonatal jaundice.

## Introduction

G6PD deficiency is the most common enzymatic disorder described in humans, with a global distribution affecting more than 400 million people [1–4]. However, its prevalence is higher in tropical and subtropical zones of the Eastern Hemisphere, such as Africa, the Mediterranean, and Southeast Asia, where it may reach up to 25%. This prevalence parallels the incidence of malaria, as there is some evidence to suggest that G6PD deficiency offers protection against severe cases of malaria [5, 6]. Approximately 11–12% of African American males are estimated to be affected by G6PD deficiency, and 24% are reported to be carriers [7]. G6PD enzyme activity is an essential component in protecting red blood cells against oxidative stress, and deficiency results in intravascular hemolysis and associated complications [8]. Over 200 G6PD variants have been described based on the genetic mutations associated with the X-linked recessive gene [6]. The World Health Organization has classified these variants according to the magnitude of the enzyme deficiency, with only class I, II, and III being of clinical significance due to the severity of hemolysis [9].

G6PD deficiency is known to be one of the risk factors for neonatal hyperbilirubinemia severe enough to cause kernicterus or jaundice-related morbidity [10, 11]. G6PD-deficient infants with severe hyperbilirubinemia often are not well correlated with hemolysis according to hematological indices, though researches using carboxyhemoglobin or end tidal CO have shown that hemolysis plays a major role in the pathophysiology of severe hyperbilirubinemia [12]. Neonatal red blood cells, which have a reduced lifespan and lower levels of enzymes like glutathione peroxidase and carbonic anhydrase, are more susceptible to oxidative damage in the presence of G6PD deficiency [13]. G6PD deficiency causing jaundice is perhaps attributed to the disruption of the oxidant-antioxidant balance and impaired recycling of peroxiredoxin 2, thus affecting bilirubin clearance [14]. It has also been reported that co-inheritance of a uridine diphosphate glucuronosyltransferase 1A1 (UGT1A1) gene variant is an additional risk factor for neonatal hyperbilirubinemia in G6PD-deficient male neonates [15].

In Qatar, screening for G6PD deficiency is routine, and it is performed on all neonates born in the country. In our hospital, we maintain a database of G6PD deficiency to provide family education and appropriate follow-up for G6PD-deficient babies. Most published studies on G6PD deficiency and neonatal jaundice have diagnosed neonatal jaundice first and then conducted G6PD deficiency testing as part of etiological evaluation, rather than as a universal test for all neonates [16]. In our study, all neonates underwent G6PD testing from cord blood, and they were followed prospectively in the jaundice clinic as part of routine well-baby follow-up. Therefore, we conducted a retrospective cohort study of all such G6PD-deficient neonates to estimate the prevalence of neonatal jaundice and its associations with various risk factors that increase the chance of neonatal jaundice.”

## Methods and materials

This retrospective cohort study encompassed all newborn babies born with G6PD deficiency between January 1, 2015, and September 30, 2022, at Al Wakra Hospital, Hamad Medical Corporation, Qatar. The primary objective of this study was to estimate the incidence of neonatal jaundice among G6PD-deficient neonates and to explore its association with various risk factors. Neonatal jaundice defined as total serum bilirubin needing phototherapy according to American Academy of Pediatrics Subcommittee on Hyperbilirubinemia 2004 [17].

### Maternal data

For mothers, we collected data on gestational age at delivery, mode of delivery, year of delivery, nationality, and place of education regarding G6PD deficiency.

### Neonatal data

Collected neonatal data included sex, birth weight, the need for phototherapy due to jaundice, peak bilirubin levels, the day of peak bilirubin, and instances of rebound hyperbilirubinemia requiring phototherapy. Laboratory data collected encompassed G6PD levels, peak serum bilirubin levels, the lowest hemoglobin levels, and the highest reticulocyte cell count.

### G6PD testing

Quantitative in vitro determination of Glucose-6- Phosphate Dehydrogenase in erythrocytes was done by using Randox G6PDH assay kit. The enzyme activity is determined by measurement of the rate of absorbance change at 340 nm due to the reduction of NADP+ by using spectrophotometer.

G6PD in RBC is released by lysing agent of reagent kit. G6PD of red cells catalyzes the glucose 6 phosphate with reduction of NADP to NADPH. The rate of reduction of NADP to NADPH is measured as increased absorbance at 340 nano meters which is proportional to the G6PD activity. Normal G6PD results varied between 339 and 500 mU/10^^9^ as per our laboratory reference.

### Follow up for G6PD deficient neonates

In Qatar, its routine to have G6PD screening test for all babies from cord blood sample. In few hours, the results usually available and if found deficient, close monitoring with transcutaneous bilirubin will be initiated. A dedicated G6PD screening team will follow up the results and educate the family regarding the results and follow up plans. American Academy of Pediatrics Subcommittee on Hyperbilirubinemia 2004 guidelines will be followed for the subsequent management and follow up of hyperbilirubinemia. When transcutaneous bilirubin is within the safe zone, babies will be discharged with follow up in a dedicated bilirubin clinic under the G6PD screening team who is maintaining a logbook of all G6PD deficient babies. Usually these babies are followed in bilirubin clinic for one week or till transcutaneous bilirubin falls within the safe zone.

### Phototherapy criteria

All neonates with G6PD deficiency identified through cord blood screening were observed in the postnatal ward for a minimum of 24 h, during which their jaundice levels were monitored using a transcutaneous bilirubinometer. Phototherapy was administered in accordance with the standard guidelines of the American Academy of Pediatrics Subcommittee on Hyperbilirubinemia from 2004 [17]. Transcutaneous bilirubin levels close to the phototherapy threshold according to the phototherapy chart, will be confirmed by serum bilirubin.

### Data collection and confidentiality

Data were extracted from our electronic documentation system, Cerner software, and collected in an Excel sheet. There was no direct interaction with human subjects, and the identity of the research subjects was not disclosed. Each patient was assigned a unique code, and all codes were securely stored in a computer system. The link between these codes and individual identifiers was deleted at the conclusion of the study, and the data will be kept in a secure locker for five years. Only the principal investigator will have access to this data, and subject identifiers will not be shared outside of the Hamad Medical Corporation.

This study adhered to the principles of the Declaration of Helsinki, Good Clinical Practice, and complied with the laws and regulations of the Ministry of Public Health (MOPH) in Qatar. It received approval from the institutional review board and ethical committee of the Medical Research Center, HMC, Qatar, with the protocol number MRC-01-23-039. Since this was a retrospective data collection study with no recruitment of subjects, informed consent was not required and was waived by the institutional review board and ethical committee. Reporting of this study followed the STROBE guidelines.

### Statistical considerations and data analysis

Anonymous data were collected and entered into a standard electronic database designed for the study and its objectives. Descriptive statistics were used to summarize demographic, laboratory, clinical, and other characteristics of both the patients and their mothers. Mean and standard deviation or frequencies and percentages were used for reporting data and results. The primary outcome variable was neonatal jaundice among G6PD-deficient neonates. Associations between two or more qualitative variables were assessed using the chi-square (χ2) test or Fisher exact test. The mean of quantitative variables between two independent groups was analyzed using the unpaired t-test. The relationship between neonatal jaundice and various risk factors such as sex, G6PD level, gestational age, birth weight, etc., was estimated by deriving adjusted odds ratios (ORs) and confidence intervals from logistic regression models. All presented P values are two-tailed, and *P* values < 0.05 were considered statistically significant. Statistical analyses were performed using the SPSS 27.0 software (SPSS Inc., Chicago, IL) and Epi-info software (Centers for Disease Control and Prevention, Atlanta, GA).”

## Results

A total of 40 305 babies were born during the study period from January 2015 to September 2022. Out of these, 1013 (2.51%) babies were G6PD deficient. Among males, the G6PD deficiency rate was 3.77% (776/20 580), and among females, it was 1.2% (237/19 725). The proportion of males among G6PD deficient babies was 76.6% (776/1013), while that of females was 23.4% (237/1013), resulting in a male-to-female ratio of 3.3:1. A total of 24.6% (249/1013) of G6PD deficient neonates received phototherapy. Other various parameters are presented in Table 1.

**Table 1:** Various maternal and neonatal parameters of G6PD deficient babies.

Parameters: (Total 1013 G6PD deficient)	Number (%)	Mean ± SD (Range)
Sex:		
Male	776/1013 (76.6%)	
Female	237/1013 (23.4%)	
Mode of delivery:		
NVD	700/1013 (69.1%)	
LSCS	313/1013 (30.9%)	
Nationality:		
Middle east	406/1013 (40.1%)	
Indian subcontinent	335/1013 (33.1%)	
African	178/1013 (17.6%)	
Southeast Asia	88/1013 (8.7%)	
Other	6/1013 (0.6%)	
Gestation age, weeks		38.6 ± 1.56 (29 – 42)
Gestation age group:		
Less than 35 weeks	27/1013 (2.7%)	
35–37 + 6 weeks	138/1013 (13.6%)	
>38 weeks	848/1013 (83.7%)	
Weight, gm		3201 ± 500 (1300 – 4700)
Weight group:		
<2500 gm	83/1013 (8.2%)	
2500–4000 gm	881/1013 (87%)	
>4000 gm	49 /1013 (4.8%)	
G6PD level, mU/10^9^ RBC, done in 92.8% (940/1013) babies		51.2 ± 55.56 (4 – 338)
G6PD level group:		
Level <30 mU/10^9^RBC	377/1013 (40.1%)	
Level >30 mU/10^9^RBC	562/1013 (59.9%)	
Phototherapy received	249/1013 (24.6%)	

*NVD* normal vaginal delivery, *LSCS* lower segmental cesarean section.

The G6PD deficiency rate varied across different nationalities of our study population: 3.8% (406/10 682) in the Middle East, 2.16% (335/16 229) in the Indian subcontinent, 1.72% (178/10 352) in African populations, and 3.46% (88/2 538) in Southeast Asian populations. In the rest of the countries, the rate was 1.19% (6/504).”

The mean G6PD levels were 51.2 mU/10^9RBC with a standard deviation of 55.56. G6PD levels in male and female babies were 34.4 ± 34.6 mU/10^9 RBC and 104.7 ± 73.3 mU/10^9 RBC, respectively, and this difference was statistically significant (*P* < 0.001). However, the need for phototherapy did not show statistical significance between the sexes; 25.7% (200/776) of male babies and 20.6% (49/237) of female babies received phototherapy (*P* value 0.11). This data is summarized in Table 2.

**Table 2:** Association of sex with G6PD level and need for phototherapy.

Parameters: total 1013 babies	Male = 776	Female = 237	Odds ratio (95% CI)/ mean difference (95% CI)	*P* value
G6PD Level mU/10^9^RBC mean ± SD	34.4 ± 34.6	104.7 ± 73.3	−70.2 (−77.3 to −63.2)	<0.001
G6PD Level group: number(%)				
<30 mU/10^9^ RBC 377	331/776 (46.2%)	46/237 (20.5%)	3.33 (2.33 to 4.76)	<0.001
>30 mU/10^9^ RBC 562	384/776. (53.7%)	178/237 (79.5%)		
Phototherapy received. Number (percentage)	200/776 (25.7%)	49/237 (20.6%)	0.75 (0.52 to 1.06)	0.11

Associations of various parameters with the need for phototherapy among G6PD deficient babies were calculated (Table 3). Statistically significant associations were found with gestational age, gestational age groups, birth weight, and birth weight groups, but no statistically significant association was observed with sex or G6PD level.

**Table 3:** Associations of various parameters with need for phototherapy.

Parameters: total 1013 babies	Phototherapy received = 249	No photo received = 764	Odds ratio (95% CI) /mean difference (95% CI)	*P* value
Sex:				
Male 776	200 (25.7%)	576 (74.2%)	0.75 (0.52 – 10.6)	0.11
Female 237	49 (20.6%)	188 (79.3%)		
MOD:				
NVD 700	173 (24.7%)	527 (75.3%)	0.97 (0.71 – 1.33)	0.882
LSCS. 313	76 (24.3%)	237 (75.7%)		
Gestation age, weeks	38.16 ± 2.01	38.8 ± 1.34	0.65 (0.43 – 0.87)	<0.001
Gestation age groups:				
<35 week 27	17 (62.9%)	10 (37%)		<0.001
35–37 + 6 week 138	54 (39.1%)	84 (60.8%)		
>38 week 848	178 (20.9%)	670 (79%)		
Weight, gm	3129 ± 575	3225 ± 473	96 (24.3 – 167.6)	0.008
Weight groups				
<2500 gm 83	30 (36.1%)	53 (63.8%)		0.033
2500–4000 gm 881	206 (23.4%)	675 (76.6%)		
>4000 gm 49	13 (26.5%)	36 (73.4%)		
G6PD level,	46.03 ± 51.8	52.9 ± 56.6	6.9 (−1.3 – 15.14)	0.100
Level group.				
<30 mU/10^9^RBC 337	100 (29.6%)	277 (73.4%)	0.858 (0.63 – 1.16)	0.319
>30 mU/10^9^RBC 562	133 (23.6%)	429 (76.3%)		
Nationality groups:				
Middle east 406	93 (22.9%)	313 (77%)		0.094
Indian subcontinent 335	87 (25.9%)	248 (74%)		
African 178	37 (20.7%)	141 (79.2%)		
south east Asia 88	31 (35.2%)	57 (64.7%)		
others 6	1 (16.7%)	5 83 (3%)		

*NVD* normal vaginal delivery, *LSCS* lower segmental cesarean section, *CI* confidence interval.

Adjusted odds for the need for phototherapy with various parameters were calculated using logistic regression analysis (Table 4). Only the gestational age group showed statistically significant odds; in the ‘gestational age <35 weeks group,’ the adjusted odds (confidence interval) were 4.03 (1.05–15.5), and in the ‘gestational age 35 to 37 + 6 weeks group,’ the adjusted odds (confidence interval) were 2.18 (1.19–3.99) compared to the ‘gestational age 38 weeks or more group’.

**Table 4:** Regression analysis: association of various maternal and neonatal factors with jaundice that received phototherapy.

Parameters: Total 1013 babies	Adjusted odds	95% Confidence interval	*P* value
SEX male	1.34	0.85 to 2.11	0.201
G6PD Level	0.99	0.99 to 1.00	0.592
G6PD level group, <30	0.97	0.67 to 1.42	0.913
Nationality groups:			0.119
Middle east	1.21	0.14 to 10.7	0.858
Indian subcontinent	1.38	0.15 to 12.2	0.768
African	1.09	0.12 to 9.83	0.933
Southeast Asia	2.34	0.25 to 21.31	0.449
Gestation age weeks	0.91	0.76 to 1.09	0.315
Gestation age groups:			0.033
<35 weeks	4.03	1.05 to 15.5	0.042
35 to 37 + 6 weeks	2.18	1.19 to 3.99	0.011
Birth weight, gm	1.00	0.99 to 1.00	0.610
Birth weight groups:			0.708
Weight <2500 gm	0.69	0.21 to 2.35	0.556
Weight 2500 to 4000 gm	0.71	0.32 to 1.60	0.413
Delivery mode NVD	1.27	0.90 to 1.79	0.167
Constant	4.42		0.689

Reference group: in sex “female”, in G6PD level group “level >30 mU/10^9^ RBC”, in nationality group “other nationality group”, in gestation age group “gestation age 38 week or more”, in weight group “weight more than 4000 gm”, in mode of delivery “LSCS” is reference group.

Out of the 249 babies who received phototherapy, 200 (80.7%) were boys, and 49 (19.2%) were girls. The mean day of onset of jaundice that required phototherapy was 2.14 days (standard deviation 1.49). Among them, 13.7% of babies experienced rebound hyperbilirubinemia once, and 4% had it twice after receiving initial phototherapy. Additional parameters are presented in Table 5.

**Table 5:** Various parameters of neonates received phototherapy.

Parameters: Total 249 babies	Mean ± SD (Range)	Number (percentage)
Sex		
Male		200 (80.7%)
Female		49 (19.2%)
G6PD level mU/10^9^RBC	45.8 ± 51.8 (4 – 331)	
Level groups:		
<30		101 (43.3%)
>30		132 (56.7%)
Birth weight, grams	3128 ± 572 (1300 – 4610)	
Gestation age, weeks	38.1 ± 2.01 (29 – 41)	
Gestation age groups:		
<35 weeks		17 (6.8%)
35–27 + 6 weeks		56. (22.4%)
>38 weeks		176 (70.7%)
Age of NNJ, days	2.14 ± 1.49 (0 – 8)	
Photo age groups:		
<24 h		11 (4.4%)
24–72 h		202 (81.1%)
>72 h		36. (14.5%)
Peak bilirubin, umol/L	261.3 ± 76.9 (98 – 616)	
Day of peak bilirubin	3.81 ± 2.84 (0 – 25)	
Rebound jaundice:		
No rebound		205 (82.3%)
Once		34 (13.7%)
Twice		10. (4%)
Hb gm/dL	16.9 ± 2.37 (9.8 – 22.1)	
Peak reticulocyte, done in 238	4.5 ± 1.91 (0.4 – 16)	

*NNJ* neonatal jaundice, *Hb* hemoglobin.

Sixteen babies had blood group incompatibility and a positive direct Coombs test. Among them, 11 babies had ABO incompatibility, 3 babies had both ABO and Rh incompatibility, one baby had Rh incompatibility alone, and one baby had minor blood group incompatibility.

Associations of sex with various parameters among babies who received phototherapy are detailed in Table 6.

**Table 6:** Association of sex with various parameters among babies who received phototherapy.

Parameters: total 249 babies	Male (201)	Female (48)	Odds ratio (95% CI)/mean difference (95% CI)	*P* value
G6PD level, mU/10^9^ RBC	35.5 ± 42.8	88.7 ± 63.6	−53.16 (−68.7 to −37.6)	0.001
Level <30 101	91 (48.4%)	10 (22.2%)	3.28 (1.53 to 7.01)	0.001
>30 132	97 (51.5%)	35 (77.8%)		
Gestation age, weeks	38.2 ± 1.88	37.5 ± 2.43	0.78 (0.15 to 1.41)	0.015
Birth weight, gram	3181 ± 558	2909 ± 584	271 (93.4 to 450)	0.003
Age of NNJ, days	2.09 ± 1.4	2.39 ± 1.8	−0.3 (−0.77 to 0.16)	0.203
Age of NNJ				
<24 h	7 (3.5%)	4 (8.3%)		0.190
24–72 h	167 (83.1%)	35 (72.9%)		
>72 h	27 (13.4%)	9 (18.7%)		
Day of peak	3.86 ± 2.83	3.62 ± 2.89	0.23 (−0.66 to 1.37)	0.607
Rebound needed phototherapy				
No rebound	162 (80%)	43 (89.5%)		0.524
Once	30 (15%)	4 (8.3%)		
Twice	8 (5%)	1 (2.1%)		
Hb, gram/dL	16.9 ± 2.45	17.16 ± 2.04	−0.24 (−1 to 0.51)	0.521
Reticulocyte count	4.4 ± 1.71	5.01 ± 2.61	−0.57 (−1.19 to 0.05)	0.072

*NNJ* neonatal jaundice, *Hb* hemoglobin, *CI* confidence interval.

Out of the 3 babies who required exchange transfusion, all were full-term male newborns. In 2015, one baby boy had a bilirubin level of 513 μmol/L on the 4th day of life and required exchange transfusion, and baby developed bilirubin encephalopathy and bilateral sensorineural hearing loss & on left cochlear implant. Another baby boy born in 2019 had a bilirubin level of 414 μmol/L on the 2nd day of life and needed exchange transfusion, clinically fine during follow up. In 2021, another baby boy had a bilirubin level of 616  μmol/L on the 4th day of life and required exchange transfusion, clinically fine till last follow up in November 2023.

## Discussion

Neonatal indirect hyperbilirubinemia (NIH) is a common problem among infants. It affects 60% of full-term and 80% of preterm newborns in the first three days of life [18]. NIH carries a substantial risk for harmful complications, including long-term neurologic impairments and death [15]. Although significant complications of NIH have become rare in recent years, even with effective therapeutic interventions such as phototherapy, bilirubin-induced neuronal damage is still a significant problem in resource-limited countries [15]. Severe NIH secondary to reduced Glucose 6-phosphate dehydrogenase (G6PD) activity is still complicated by kernicterus, which is a serious neurological disease [4, 15, 19].

The global prevalence of G6PD deficiency was estimated to be 4.9% [5]. There is a wide geographical distribution of G6PD deficiency, with the highest prevalence reported across sub-Saharan Africa [20]. In our study, the incidence of G6PD deficiency was 2.51 per 100 live births. Among them, males accounted for 76.6%, and females for 23.4%.

G6PD deficiency is an X-linked recessive disease. In our study, we detected a male-to-female ratio of 3.3–1. A similar ratio was reported by other studies [19, 21], and interestingly, the rate of neonatal jaundice needing phototherapy was also not significantly lower in females than in males; 20.6% and 25.7% in females and males, respectively. The mean G6PD level was 51.2 mU/10^9 RBC with a standard deviation of 55.56. G6PD levels of male and female babies were 34.4 ± 34.6 mU/10^9 RBC and 104.7 ± 73.3 mU/10^9 RBC, respectively, and were statistically significant (*P* < 0.001). However, the need for phototherapy was not statistically significant between sex groups.

G6PD deficiency is a major risk factor for neonatal hyperbilirubinemia. Multiple reports have shown that G6PD-deficient infants are predisposed to neonatal jaundice [10, 11]. In a study conducted on African American neonates, among G6PD-deficient neonates, the rate of phototherapy was 20.3% compared to control subjects at 5.7% (relative risk: 3.53; 95% confidence interval: 1.91–6.56) [22]. In our study, about 25% of G6PD-deficient neonates developed jaundice needing phototherapy.

We found that gestational age, gestational age groups, birth weight, and birth weight groups had a statistically significant association with the need for phototherapy among G6PD-deficient babies (Table 3). No statistically significant association with sex or G6PD level was observed. We calculated adjusted odds for the need for phototherapy with various parameters using logistic regression analysis and found that only the gestational age group had statistically significant odds. In the ‘gestational age <35 weeks group,’ the adjusted odds (confidence interval) were 4.03 (1.05–15.5), and in the ‘gestational age 35 to 37 + 6 weeks group,’ the adjusted odds (confidence interval) were 2.18 (1.19–3.99) in comparison to the ‘gestational age 38 weeks or more group’.

The mean day of onset of jaundice that needed phototherapy was 2.14 days, with a standard deviation of 1.49. 13.7% of babies had rebound hyperbilirubinemia once, and 4% had it twice after receiving initial phototherapy. In the study published by Atay et al., the day of onset of jaundice was 6.1 ± 3.7 days [19]. In our study, the detection of jaundice needing phototherapy was earlier; this may be due to the routine early follow-up of all high-risk neonates in the dedicated jaundice clinic. The mean peak bilirubin was 261.3 ± 76.9 μmol/L, and the mean day of the peak was 3.81 ± 2.84. Hemoglobin was 16.9 gm/dL ± 2.37, and the reticulocyte count was 4.5% ± 1.91 among babies that needed phototherapy. Other study results are comparable to the study from demographically similar Bahrain, in which hemoglobin was 16.4 gm/dL and reticulocyte count was 3.3% [11].

G6PD deficiency can cause severe neonatal indirect hyperbilirubinemia, which may lead to kernicterus or bilirubin encephalopathy [16, 23]. In a study from Oman, 71% of kernicterus patients were reported to be due to G6PD deficiency [24]. In our study, only 3 babies (0.29%, 3/1013) needed exchange transfusion and one of them had bilirubin encephalopathy. The routine screening of G6PD and subsequent close follow-up of G6PD-deficient neonates helped implement early phototherapy, thus reducing the need for exchange transfusion and preventing bilirubin-induced neurologic complications in Qatar.

## Conclusion

G6PD deficiency is a major risk factor for neonatal hyperbilirubinemia and bilirubin-induced neuronal damage. Early detection and intervention with phototherapy are simple, cost-effective strategies to identify infants at risk, monitor and treat hyperbilirubinemia effectively to prevent kernicterus. Our study demonstrates that this model of routine G6PD screening and routine follow-up could reduce the need for exchange transfusion and help prevent chronic sequelae of hyperbilirubinemia associated with G6PD deficiency.

Being a retrospective study, the findings of this study should be evaluated carefully. In this study, we calculated associations of various parameters with the need for phototherapy among G6PD-deficient babies. We found that gestational age, gestational age groups, birth weight, and birth weight groups had a statistically significant association with the need for phototherapy, but in regression analysis, only the gestational age group was found to be statistically significant. No statistically significant association with sex or G6PD level was seen.

## Data Availability

Deidentified data will be provided as per request from journal.
